# Assessing the role of protected areas in the land-use change dynamics of a biodiversity hotspot

**DOI:** 10.1007/s13280-023-01886-5

**Published:** 2023-06-01

**Authors:** Marcelo Henrique Schmitz, Edivando Vitor do Couto, Erick Caldas Xavier, Leonardo da Silva Tomadon, Rodrigo Pedro Leal, Angelo Antonio Agostinho

**Affiliations:** 1 Departamento de Biologia, Programa de Pós-Graduação em Ecologia de Ambientes Aquáticos Continentais - PEA, Universidade Estadual de Maringá, Maringá, Paraná 87020-900 Brazil; 2 Technische Universität München, Arcisstraße 21, 80333 Munich, Germany

**Keywords:** Conservation, Effectiveness, Geoprocessing, Human impacts, Land-use policy, Wetlands

## Abstract

Although protected areas (PAs) are designed to safeguard natural ecosystems from anthropic modifications, many PAs worldwide are subjected to numerous human-induced impacts. We evaluated whether the establishment of PAs in the Upper Paraná River floodplain region could reduce anthropic landscape changes and whether there is a difference in protection when using different PA restriction categories. We analyzed the overall landscape dynamics using 30 years of land-use time series data and evaluated the change intensity via a partial land-use intensity analysis. Despite the increasing landscape anthropization, the PAs seemed to relieve the general change process, protecting natural areas mainly from agricultural expansion. Concerning the degree of use restriction, more restricted protection led to less human-induced changes. Finally, accessing PA effectiveness is a multidisciplinary challenge for researchers; however, this knowledge is crucial to avoid misunderstandings or poorly crafted public policies or decisions that may harm the environment.

**Supplementary Information:**

The online version contains supplementary material available at 10.1007/s13280-023-01886-5.

## Introduction

Anthropic landscape changes are the major drivers of harmful environmental impacts such as deforestation, global biodiversity declines, and disruptions in the ecosystem structure and functioning (Vitousek et al. 1997). These changes in the landscape are dynamic and occur on distinct spatiotemporal scales (With et al. 1997). Typically, the most economically productive lands, which are flat, highly fertile, or around cities are the first to be altered; simultaneously, the remnants of original vegetation are usually in less productive areas or represent the most challenging areas to access (Silva et al. 2007). This pattern of occupation has severe consequences on habitat connectivity and species distribution (With et al. 1997; Silva et al. 2007; Tomadon et al. 2019). Therefore, identifying and quantifying land-use changes are crucial to understand the complex dynamics of human effects on the landscape (Aldwaik and Pontius 2012).

Furthermore, rather than simply analyzing landscape transformations over time, it is more informative to analytically evaluate systematic processes in the landscape (Aldwaik and Pontius 2012; Quan et al. 2019). In this context, it is important to determine the intensity of land-use changes, which indicates if the landscape of a given area at a given time interval changed at a fast or slow pace (Aldwaik and Pontius 2012). The importance of this information lies in the counter-measures that can be adopted to protect areas from fast anthropogenic land-use changes, especially in vulnerable ecosystems such as natural forests, wetlands, and others (Malekmohammadi and Jahanishakib 2017).

It is widely known that protected areas (PAs) serve essential roles in species conservation (Oliveira et al. 2016; Tomadon et al. 2019), the maintenance of freshwater ecosystems (Abell et al. 2007; Bailly et al. 2021), the conservation and maintenance of indigenous communities and cultures (Zurba et al. 2019), the restoration of degraded ecosystems (Porter-Bolland et al. 2012), carbon sequestration (Melillo et al. 2015), and safeguarding natural ecosystems from anthropic modifications (Metzger et al. 2019). Furthermore, when compared to less restrictive PAs, those with higher use restrictions perform better in terms of environmental protection, such as protecting against fires (Jesus et al. 2020) or forest loss (Leberger et al. 2020). Despite this, many PAs worldwide have been subjected to several impacts due to anthropic actions. Some of the most commonly reported examples include illegal logging (Moretti et al. 2020), fires to change land use (Berlinck and Batista 2020), the hunting and trafficking of wildlife (Ahmad Zafir et al. 2011), and crop cultivation, which commonly occur in and around PAs and result in overall landscape change and habitat fragmentation (Fearnside 2001; Silva et al. 2007).

To protect its high biodiversity, Brazil created the National System of Nature Conservation Units (using the acronym SNUC in Portuguese) in the year 2000 through law nº 9985, which establishes criteria and norms for the creation, implementation, and management of PAs. In this system, PAs are planned based on their ecological relevance and grouped according to their respective management objectives. The grouping is presented as follows: (i) Integral Protection Areas designed to preserve nature, in which only the indirect use of resources is allowed, and activities involving consumption, collection, or damage are prohibited; (ii) Sustainable Use Areas that aim to make nature conservation compatible with the sustainable use of part of the area’s natural resources. The Brazilian system was based on the system proposed by the International Union for Conservation of Nature (IUCN) and (i) is similar to IUCN category II, where the land use is more restricted and (ii) is analogous to IUCN categories V and VI, with fewer use restrictions (IUCN 1994). Consequently, the increase in the number of Brazilian PAs over the last two decades has played a key role against the decline of biodiversity, protecting thousands of species, of which many remain unknown due to low investment in sampling efforts, especially in newer PAs (Oliveira et al. 2017).

However, Brazil faces a series of obstacles to protecting its biodiversity. First, unsuitable political decisions are continuously being made that have negative implications for the environment (Azevedo-Santos et al. 2017, 2020; Alves et al. 2019; Metzger et al. 2019; Lima et al. 2020; Conceição et al. 2022) and aim to reduce the size and degree of PA protection (Alves et al. 2019; Metzger et al. 2019). One of the most controversial episodes was the approval of new forest legislation in 2012 (Brazilian law No. 12.651/2012) that reduced the legal protection of Brazil’s biomes and resulted in high deforestation rates (Abessa et al. 2019). Although the Amazon rainforest is currently the most prominent front of Brazilian deforestation, the two most anthropized Brazilian biomes are the Atlantic Forest and the Cerrado (Mittermeier et al. 2004). As a result, these two biodiversity hotspots suffer intense and recurring anthropic impacts despite having high levels of endemic fauna and flora and being crucial for biodiversity conservation (Rezende et al. 2018; Tomadon et al. 2019).

Additional limitations for conservation in developing countries can include a lack of reliable data on the distribution of biodiversity components, ecosystem services, alternative land-use methods, and their costs (Di Minin et al. 2017), and the severe underfunding of the PAs (Silva et al. 2021). Furthermore, these areas are unevenly distributed, and insufficient to protect biodiversity (Oliveira et al. 2017). Also, low levels of investment in PAs undermine the capacity for sampling and inventorying biodiversity in active PAs, which may produce incomplete information about biodiversity protection and composition and ultimately cast doubts on the general effectiveness of PAs (Oliveira et al. 2017). However, given the scarcity of investments, new datasets associated with low-cost methods, the integration of remote sensing into in situ monitoring system networks, as well as the use of landscape metrics—which do not necessarily require field-generated data—are becoming increasingly available for identifying priority areas for conservation actions, as well as for monitoring biodiversity (Di Minin et al. 2017).

This study aims to investigate the effects of the establishment of PAs in terms of reducing or mitigating changes in land use caused by anthropogenic actions in the landscape and to assess whether there is a difference in protection according to the degree of use restriction of the selected PAs. To achieve this, we analyzed a 30-year land-use time series of the region of the Upper Paraná River floodplain, which is considered a biodiversity hotspot and hosts three PAs with different legal use restrictions. The general land-use dynamics were analyzed considering the years before and after the creation of the PAs with different degrees of protection, while the overall land-use intensity was obtained through a partial land-use intensity analysis. This study contributes to scholarly efforts that investigate and produce data for developing effective strategies to protect biodiversity and the environment, particularly in developing countries where they are most needed.

## Materials and Methods

### Characterization of the study area

**Fig. 1:**
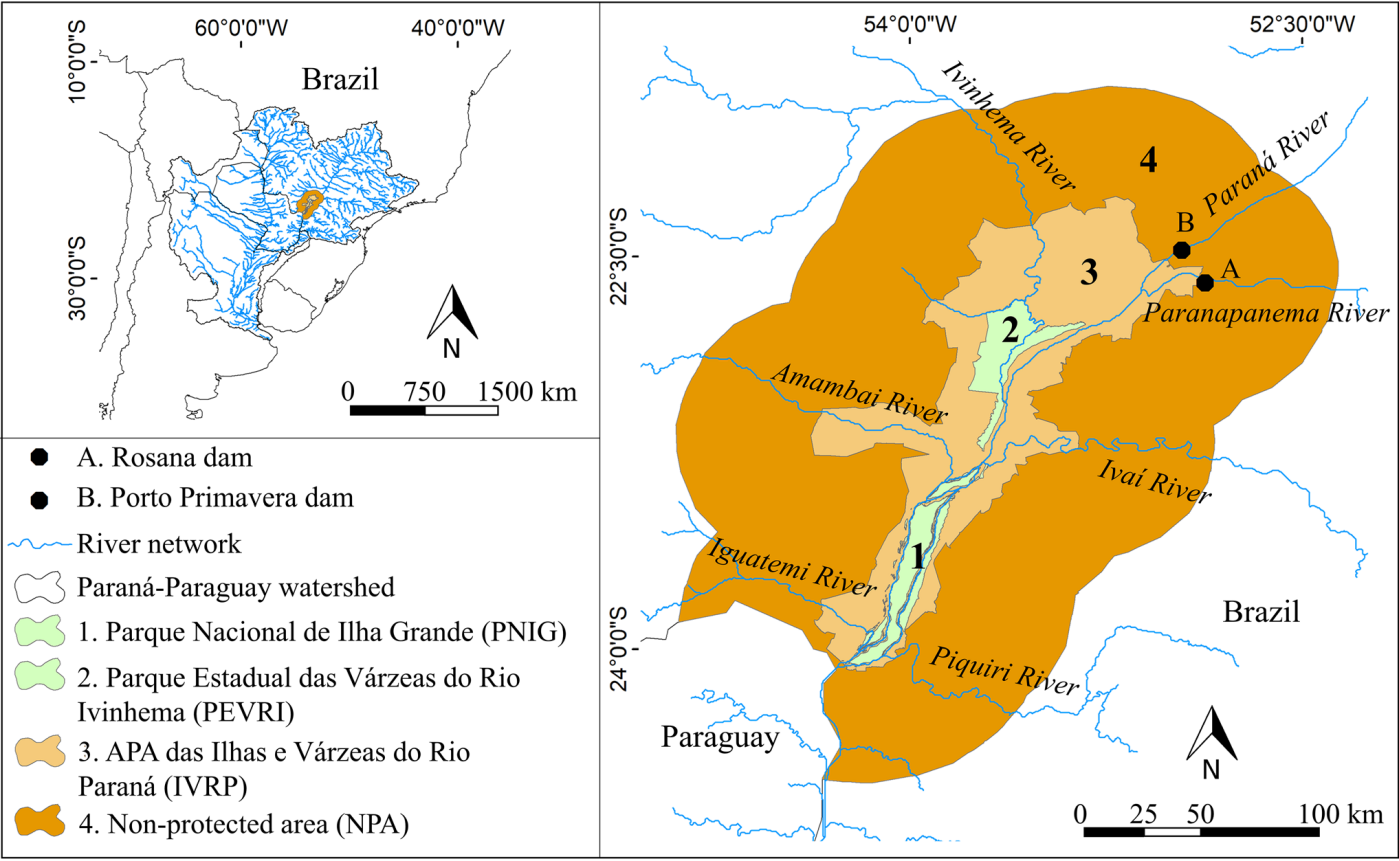
Map of the study area. The Integral Protection Areas PNIG (1) and PEVRI (2) are represented in green. The IVRP (3) Sustainable Use Area is represented in orange. The Non-Protected Area (4—dark orange) was extracted with a buffer of 50 km from the IVRP edges

This study was conducted in three PAs in the Upper Paraná River floodplain: (i) Área de Proteção Ambiental das Ilhas e Várzeas do Rio Paraná (IVRP hereafter); (ii) Parque Nacional de Ilha Grande (PNIG); (iii) Parque Estadual das Várzeas do Rio Ivinhema (PEVRI) (Fig. 1). The Upper Paraná River floodplain is located in the transition zone between the Atlantic Forest biome (seasonal semideciduous forest, State of Paraná) and the Cerrado biome (State of Mato Grosso do Sul), where floodplains and riparian forests are typical elements of the landscape (Agostinho et al. 2007). Currently, this region comprises the last stretch of the Paraná River to be free of dams in Brazilian territory, highlighting the crucial relevance of protecting critical archaeological sites and many species of fauna and flora, which are priority targets for conservation (Agostinho et al. 2007).

The creation of the PNIG Integral Protection Area resulted from a compensatory measure to protect the Paraná River floodplain, whose biota was affected by the Itaipú reservoir, which invaded part of the former National Park of Sete Quedas in 1982 (Brasil 1997a; ICMBio 2008). PNIG has an area of 76,138.19 ha and was created in September 1997. Historically, PNIG was essentially an archipelago of public areas and had its margins occupied by small farmers from different Brazilian states, while its innermost part was used by local farmers that used large areas of the plain for livestock production (Campos 2001; Xavier 2015).

In turn, PEVRI was created as a compensatory measure for constructing the Porto Primavera reservoir (Engenheiro Sérgio Motta hydroelectric plant) by subtracting the upper half area of the former floodplain area (Mato Grosso do Sul 1998). Also categorized as an Integral Protection Area, PEVRI has an area of 73,345.15 ha and was created in December 1998. PEVRI was formed by a mosaic of livestock farms, which were expropriated when the PA was created (Campos 2001; Xavier 2015).

Finally, IVRP, which is categorized as a Sustainable Use PA, was created in September 1997 and has an area of 1005,180.71 ha (Brasil, 1997b). IVRP also went through the same process of occupation as the aforementioned PAs. In the external area, the occupation was similar to what occurred with PEVRI, while its islands experienced a process similar to that conducted in PNIG (Rosa 1997; Xavier 2015). IVRP covers the two others Integral Protection PAs. Notably, one of the objectives for the delimitation of the surrounding Sustainable Use Area was to create a buffer zone for these two PAs (Xavier 2015). The three PAs were targets of only a few small restoration actions, and practically all the forest restoration in these areas occurred naturally (Xavier 2015).

The PAs share a history of degradation and disorderly occupation of their landscape. The process involved the extraction of noble wood, followed by the clearing of the forest, with the subsequent use of fire to promote the removal of stumps and roots, which is necessary for the conversion to pasture (Rosa 1997). This process was frequently accompanied by the invasion of public areas, known as “grilagem”—a method that is currently used in the Amazon biome (Nogueira and Lima 2018).

Also, the PAs continue to be affected by the presence of two invasive grass species (*Urochloa arrecta* (Hack. ex T.Durand & Schinz) Morrone & Zuloaga and *Megathyrsus maximus* (Jacq.) B.K.Simon & S.W.L.Jacobs) (Thomaz et al. 2009) that were previously used for livestock and are highly competitive and very resistant to disturbances, thereby hindering the regeneration of native vegetation (Bianco et al. 2015; Leal et al. 2022).

**Fig. 2:**
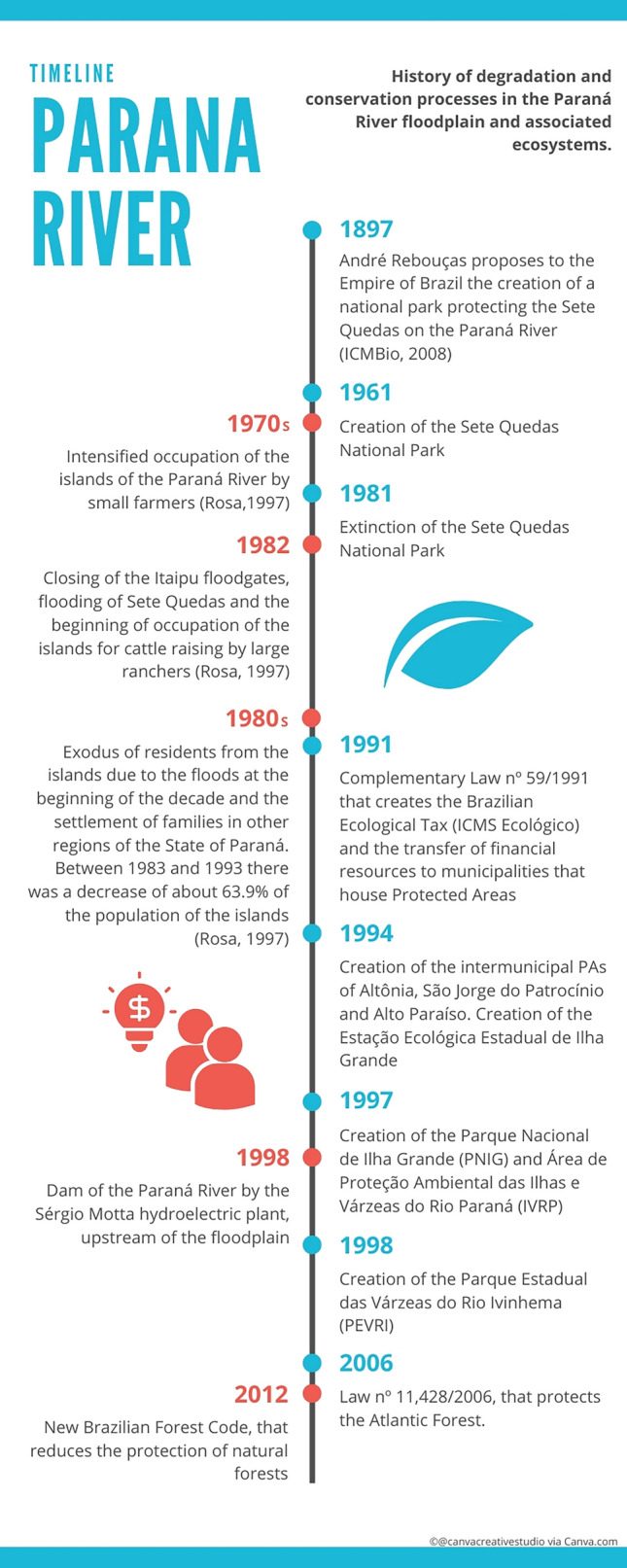
Timeline summarizing the main historical events that may have affected the Paraná River floodplain

Twenty-five Brazilian municipalities have part of their territories within the aforementioned PAs. The Brazilian demographic survey—carried out by the Brazilian Institute of Geography (IBGE, 2023) between the years 1990 and 2010—estimated a population of about 990,788 in the studied PAs in the year 1990. In 2010, the population increased to 1,071,553, representing a 1.08% increase (Supplementary Material S1). A timeline summarizing the main historical events that may have affected the Paraná River floodplain is presented in Fig. 2.

### Case study design

For our analyses, we considered a “degree of protection” factor with three levels: Integral Protection Area (IPA), Sustainable Use Area (SUA), and Non-Protected Area (NPA). The IPA category was represented by the combined areas of the PNIG and PEVRI. We chose to group PNIG and PEVRI based on the assumption that these areas are close and subjected to very similar land-use processes throughout time. Indeed, specific analyzes comparing the main patterns of PNIG and PEVRI corroborated their similarity (Supplementary Material S2 and S3). The SUA category was represented by IVRP, excluding the areas of PNIG and PEVRI. To define the NPA, we first calculated the average width of the IVRP, which resulted in a value of 47 km. Thus, we selected a 50 km buffer from the edges of the IVRP shapefile as representative of the NPA (Fig. 1). The NPA considered only Brazil, excluding the southwestern portion that belongs to Paraguayan territory and, in turn, is subjected to different territorial laws than those of Brazil. Finally, we considered 1998 as the creation year of the three PAs.

Thirty land-use maps from between 1989 and 2018 were analyzed. All the land-use rasters had the same matrix resolution (30 × 30 m), the same extension, and EPSG 6933 (WGS 84/NSIDC EASE-Grid 2.0 Global). We used data from MapBiomas collection 5. The MapBiomas project is a multi-institutional initiative to generate land cover and use maps. MapBiomas collection 5 classifies 33 land-use categories using an empirical decision tree classification algorithm based on single-date spectral mixture analysis. The complete description of the project and classification processes can be found at http://mapbiomas.org (Projeto MapBiomas, 2023).

The original 33 categories of the MapBiomas mapping were reclassified into nine equivalent categories. The grouping of the categories was conducted to maintain the maximum possible consistency between the categories of land use. The categories were as follows: Natural Forest, Forest Plantation, Natural Non-Forest, Agriculture; Pasture, Non-Vegetated Area, Urban Infrastructure, Water, and Wetland.

To analyze the landscape dynamics in the years before and after the creation of the PAs while considering the aforementioned degrees of protection, we grouped the 30 land-use rasters while considering three ten-year time intervals (1989–1998, 1999–2008, and 2009–2018) to produce three products for each time interval: (i) change intensity maps highlighting the total amount of land-use transitions in the studied area; (ii) bar plots to quantify these transitions for each degree of protection; (iii) a chart highlighting the net/gross losses and gains of all the land-use categories. As described by Aldwaik and Pontius (2012), we also used the 30-year time series to apply the interval level of a land-use intensity analysis and identify the temporal intensity of land-use changes in the R package OpenLand (Exavier and Zeilhofer 2020).

The intensity analysis interval level assesses the change intensity by determining on which time intervals the overall annual rate of change is fast or slow (Aldwaik and Pontius 2012). It is performed by analyzing the total change in each time interval to examine how the size and annual rate of change vary across time intervals. After the calculation of the annual change intensity for each time interval, the analysis compares the observed rates to a uniform rate that would exist if the annual changes were distributed uniformly across the entire time extent (Aldwaik and Pontius 2012).

**Fig. 3:**
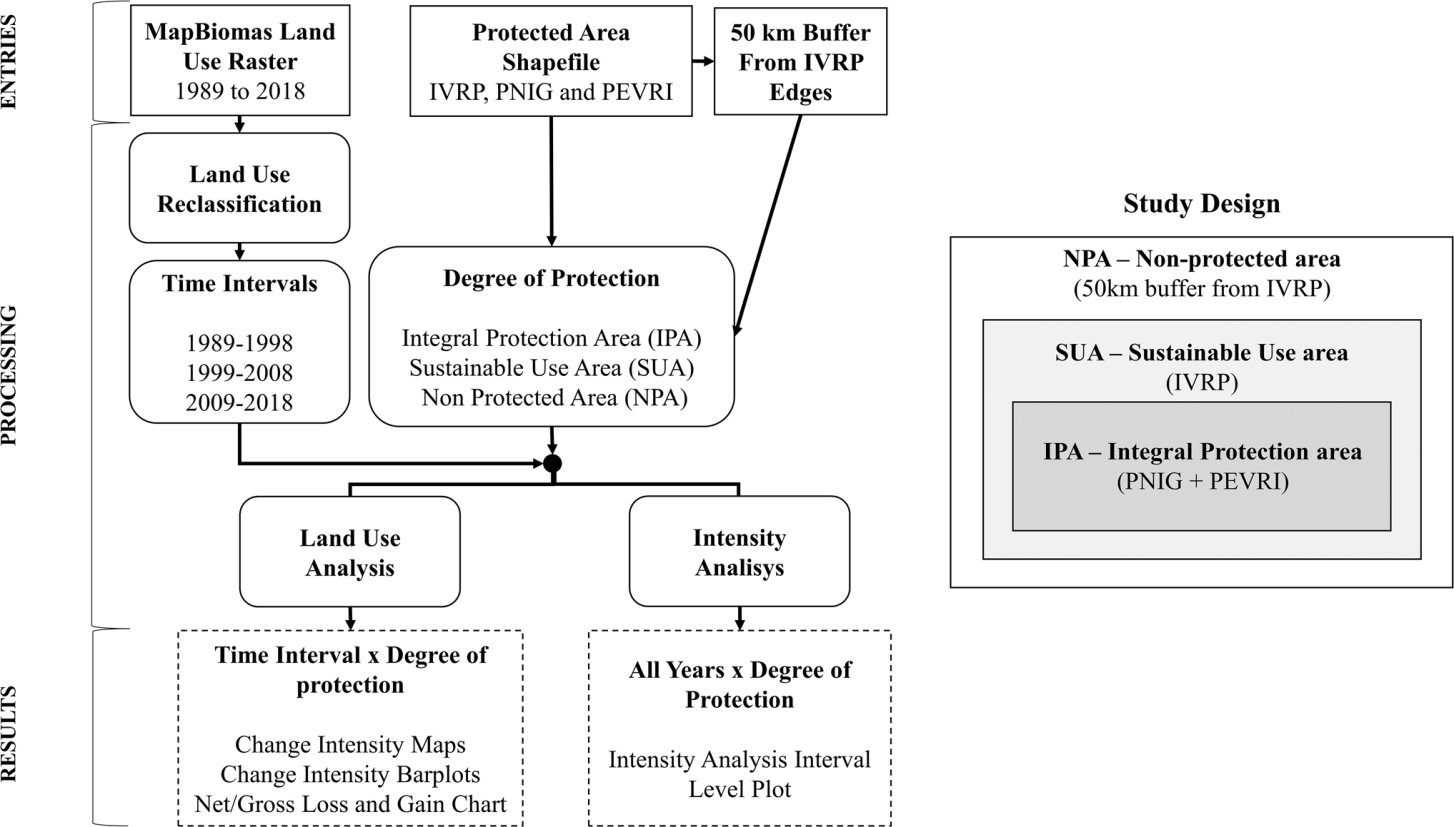
Conceptual model of the research design. IVRP: Área de proteção Ambiental das Ilhas e Várzeas do rio Paraná; PNIG: Parque Nacional de Ilha Grande; PEVRI: Parque Estadual das Várzeas do Rio Ivinhema

The shapefiles of the PAs were obtained through the website of the Brazilian Ministry of the Environment (MMA, 2023). The hydrography shapefile was obtained through the HydroSHEDS project website (HydroSHEDS 2023). All maps were created in QGIS version 3.10.11 software. The conceptual model describing the research design is presented in Fig. 3.

## Results

### Overall change

**Fig. 4:**
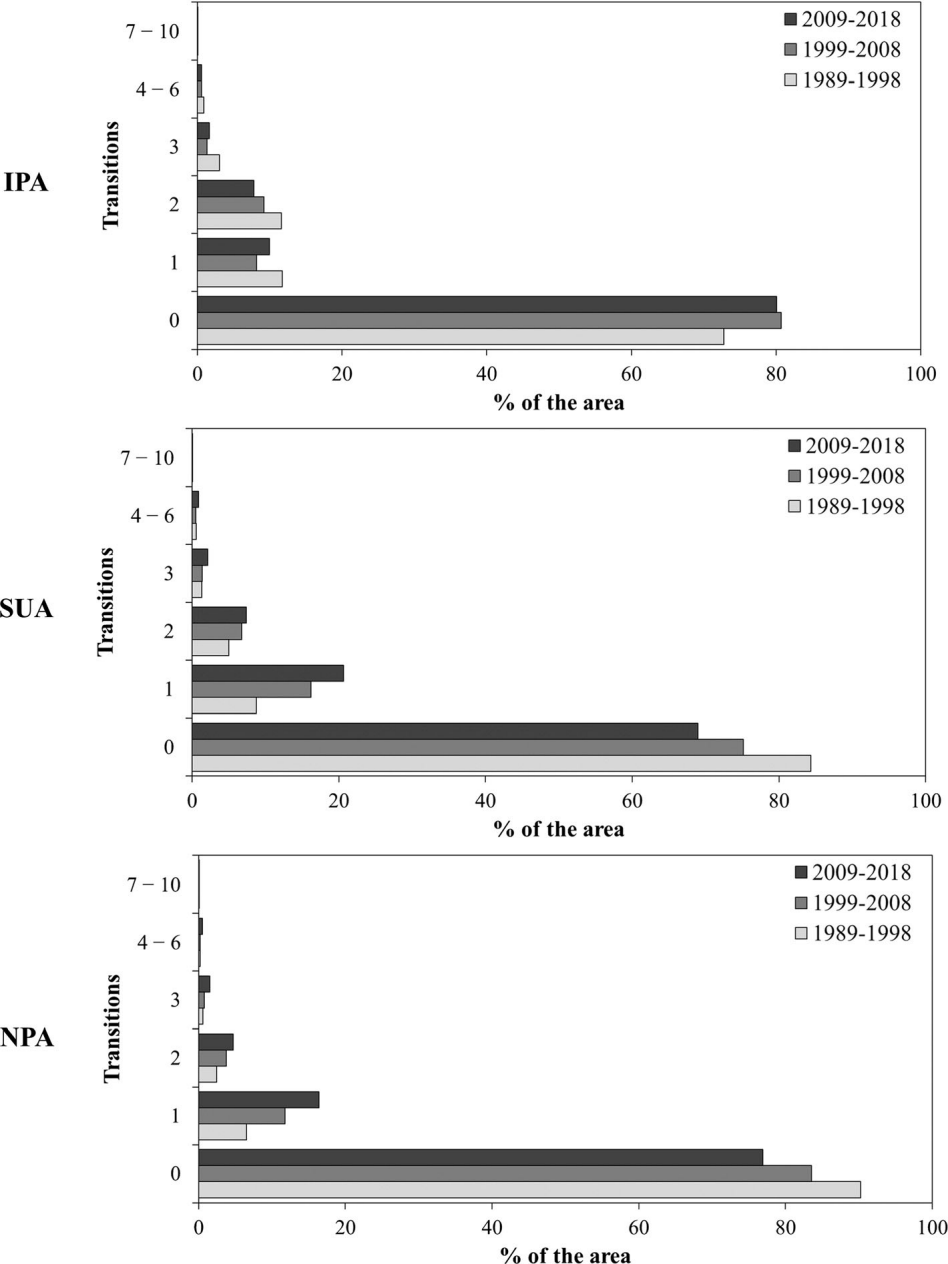
Bar plots of the accumulated transitions during the periods of 1989–1998 (light gray), 1999–2008 (regular gray) and 2009–2018 (dark gray) for the three studied areas: *IPA* Integral Protection Area, *SUA* Sustainable Use Area, *NPA* Non-Protected Area

The accumulated land-use transitions are represented in the charts shown in Fig. 4. From 1989 to 1998, the IPA presented fewer zero transitions than in 1999–2008 and 2009–2018. On the other hand, the other transitions were higher in 1989–1998 than in the other two periods. The SUA and NPA presented similar patterns for the accumulated transitions. However, contrary to the IPA, the SUA and NPA presented a higher number of zero transitions in 1989–1998, and higher transition values in 1999–2008 and 2009–2018. Also, different from the pattern of the IPA, the SUA and NPA showed an increase in the transitions occurring in 1999–2008, and even more in 2009–2018. The change intensity maps with the spatialization of land-use transitions are provided in Supplementary Material S4.

**Fig. 5:**
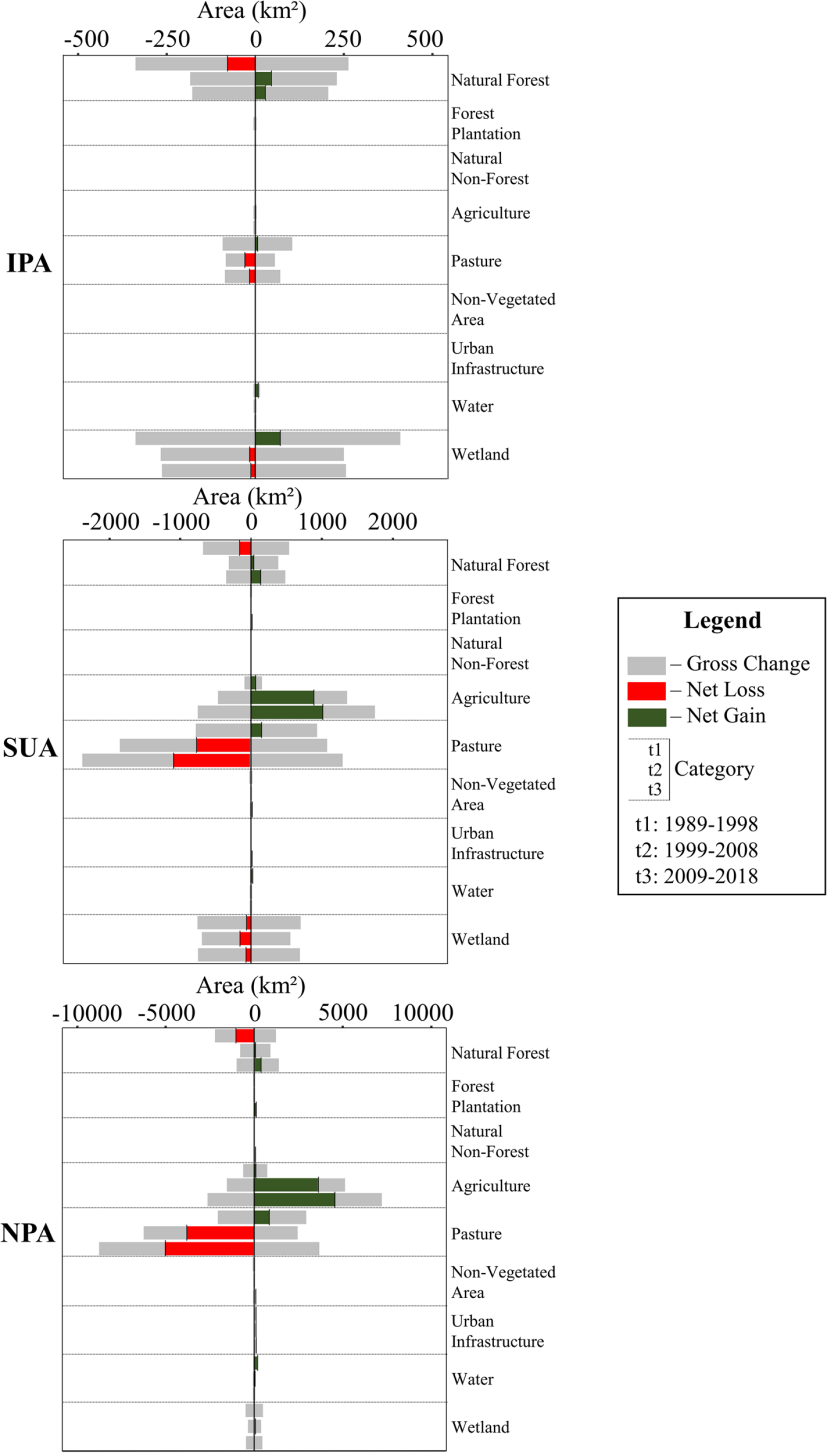
Net gains (green), losses (red) and gross change (gray) of the land-use categories for the *IPA* Integral Protection Area, *SUA* Sustainable Protection Area, and *NPA* Non-Protected Area, during the three intervals: 1989–1998 (left bar), 1999–2008 (center bar) and 2009–2018 (right bar)

The gross change and net gains and losses are shown in Fig. 5 for each land-use category. Upon analyzing the plot at the top of Fig. 4, the IPA presented a pattern change from a net loss to a net gain of the Natural Forest category from 1989–1998 to 1999–2008 and 2009–2018, while Pasture and Wetland lost area from the first to the other time intervals. The middle plot represents the SUA dynamics, in which Natural Forest also changed from losing to gaining area after 1998. Also, in the SUA, the Agriculture category showed a large increase in area, especially during the 1999–2008 and 2009–2018 periods. On the other hand, the Pasture category changed from a small net gain in 1989–1998 to significant net losses in 1999–2008 and 2009–2018. Also, the Wetland category presented regular net losses at all intervals.

The bottom plot of Fig. 4 shows the landscape dynamics for the NPA. In this plot, the pattern was similar to what happened for the SUA, with Natural Forest changing the net losses of 1989–1998 to net gains in the other two intervals. Also, Agriculture presented high values of net gain in the two last intervals, while Pasture changed from gaining area to losing area after 1998.

### Intensity analysis—Interval level

**Fig. 6:**
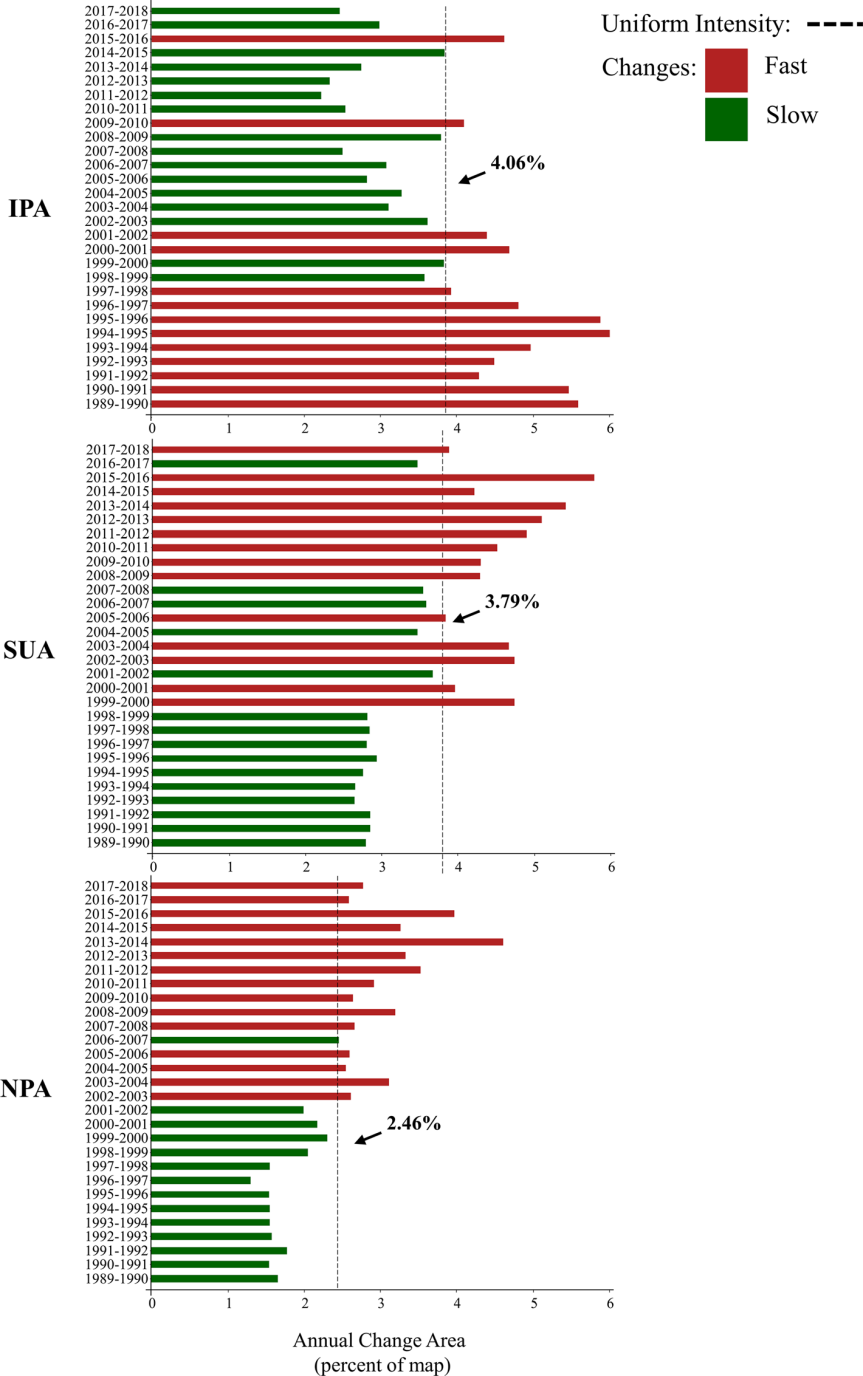
Results from the interval level of the intensity analysis. Green bars indicate slow changes and red bars indicate fast changes. *IPA* Integral Protection Area, *SUA* Sustainable Use Area, *NPA* Non-Protected Area

The results of the interval level of the land-use intensity analysis are presented in Fig. 6. The IPA showed the highest uniform intensity (U) of change of the three PAs (U = 4.06% per year). Continuous fast annual changes occurred from 1989–1990 until 1997–1998. Moreover, slow annual changes started continuously appear in 2002–2003 and remained until the last time interval, except for 2009–2010 and 2015–2016.

For the SUA, U was equal to 3.79%, and the landscape dynamics changes showed an inverse pattern when compared to the IPA. At the SUA, slow annual changes occurred from 1989–1990 until 1998–1999. From 1999–2000 to 2007–2008, landscape changes alternated between fast and slow. Finally, from 2008–2009 to the last interval, fast changes occurred, except during the 2016–2017 interval. In the NPA, U was equal to 2.46% per year. The NPA results showed a pattern similar to that found in the SUA, with slow changes occurring from 1989–1990 to 2001–2002 and shifting to fast changes from 2002–2003 until 2017–2018, except for 2006–2007.

## Discussion

Between 1998 and 2002, the three evaluated areas showed a shift in forest and farming dynamics, in which the IPA exhibited forest recovery and a decrease in pasture areas, while the SUA and NPA also presented forest recovery and an increase in agriculture. It seems that the PAs’ implementation softened the general process of landscape change, while protecting the natural categories from anthropic expansion mainly represented by an increase in farming activities. In this dynamic, the degree of protection played an essential role, where the IPA (with stricter protection) exhibited fewer landscape changes and change intensity when compared to the SUA and NPA.

The creation of buffer zones around PAs is an approach that has been proposed to conciliate the protection of biodiversity and the human occupation of the surroundings (Freitas-Lima and Ranieri 2018). It resembles the well-known land-sharing versus land-sparing mechanism (Green et al. 2005). In our case, the SUA, which was created to serve as an impact smoothing buffer for the IPA, has policies that follow the land-sharing concept, as a multifunctional landscape that serves both conservation and agricultural purposes, ultimately allowing the land sparing of the parks that compose the IPA (Green et al. 2005; Vongvisouk et al. 2016). Since land use around PAs is developed without environmental planning in most situations, the adoption of a buffer zone aims to smooth the general anthropic impacts on nature, thereby allowing the assessment of current and future threats for the PAs (Freitas-Lima and Ranieri 2018). Our results demonstrate that the creation of the IPA enhanced landscape stability, as shown by fewer transitions between categories after 1998. Thus, despite the increasing anthropization of the studied area (mainly due to farming activities), the land-sparing policies seemed to favor the landscape by diminishing the overall anthropic change intensity and favoring forest recovery in the IPA.

Moreover, a major shift in the landscape change pattern was observed between the years 1998 and 2002. The IPA began to change more slowly, with natural forests not being reduced and starting to show area gains. Since there were no considerable reforestation measures in parks composing the IPA (Xavier 2015), the observed increase in vegetation cover may be related to a dampening effect arising from the creation of the PAs, possibly based on their stricter land use resulting in greater monitoring in the region and an increase in widespread environmental awareness, ultimately leading to less deforestation and habitat loss (Pfaff et al. 2014; Jesus et al. 2020).

In contrast to the IPA, the SUA and NPA presented an inverse pattern, with the landscape being more unstable over time and showing increasing farming activities. The effect of PA land-use restrictions over their neighboring areas can take two general forms: leakage, when the landscape changes that would occur inside the PA are halted by stricter land-use regulations and relocated to a neighboring area; blockage, when a positive spillover effect from the PA results in less land-use changes than would have otherwise occurred in the unprotected surroundings (Fuller et al. 2019; Guerra et al. 2019). In our study, the SUA and NPA seemed to experience a blockage effect from the IPA, resulting in the increase of agriculture, reduction of pasture, and a shift from losing to gaining forest area after 1998. In the specific case of the SUA, the shift from pasture to agriculture, along with the recovery of the forest areas, demonstrates an apparent successful application of the land-sharing perspective by conciliating the protection of biodiversity and human occupation of landscape (Green et al. 2005).

Moreover, the massive loss of pasture areas in the SUA and NPA can be associated with a shift from cattle cultivation to crops since farming activities alternate over time based on the prices of local and global markets (Rudke et al. 2019). Notably, in anthropic landscape changes, the most productive areas with flat land are typically the first to be altered (Silva et al. 2007), making the pasture areas more suitable to convert to crops than the legally protected forests. The expansion of farming areas over time is also reflected by the high values of net area gains from the agriculture category, especially since the 2000s. Nevertheless, despite the recovery of forest area, the indication of agricultural growth in the SUA raises some concerns, especially regarding the cultivation of soybean and sugarcane, which are very common in the region (Rosa 1997; Xavier 2015). There is evidence pointing to negative environmental impacts caused by these crops, such as deforestation (Lima et al. 2020), the invasion of exotic species and loss of diversity (Pozebon et al. 2020), and the pollution of freshwater ecosystems (Santos and Esteves 2015). Moreover, the hazardous effects are not limited to the land directly converted to the fields since the “dragging effect” of human infrastructure caused by these crops results in additional damage to the environment (Fearnside 2001).

In contrast to the pattern observed in the IPA, the landscapes of the SUA and NPA began to change at higher rates in the 2000s. In contrast to the restricted laws of the IPA, the general land-use restrictions in the SUA seemed insufficient to contain some of the anthropic changes. Also, a possible leakage effect (Fuller et al. 2019), caused by the creation of the IPA, may have enhanced the search for land in the SUA and NPA after 1998. The region comprising Paraná and Mato Grosso do Sul states is considered the largest agribusiness hub of Latin America (Couto et al. 2020). Nonetheless, catalysts such as the modernization of agriculture, government subsidies for the cultivation of soybean and sugarcane, and a favorable global economy for agribusiness have made the Cerrado the new Brazilian agricultural frontier during that period (Gomes et al. 2019), which may have contributed to the accelerated occupation of the SUA and the surrounding NPA.

The wetland areas of the Upper Paraná River floodplain are composed of numerous secondary channels, connected and isolated lakes, and the main channels of the Paraná, Bahia, and Ivinhema rivers. The natural flood regime is responsible for changes in the landscape, especially transitions between wetland areas, forests, and non-vegetated areas (Souza Filho and Fragal 2013). In years of intense flooding, a large part of the riparian vegetation is submerged and replaced first by non-vegetated areas when the water recedes, and then by plants of lower successional stages (Souza Filho and Fragal 2013). The natural flooding process provides an abundance of resources for fish species, favors the reproduction of migratory species, and increases the dispersal of organisms (Quirino et al. 2017; Oliveira et al. 2020). Despite its importance, this natural dynamic is constantly threatened by anthropic activities that change the land use and cover (Yofukuji et al. 2023) and by the construction of hydroelectric plants, which is known to cause several harmful environmental impacts (Pereira et al. 2017; Oliveira et al. 2020). The immediate landscape changes caused by the damming of rivers, especially the construction of the Rosana, Taquaruçu and Porto Primavera dams upstream of the studied area and objectively outside of the PAs may have been responsible for the reduction of wetland areas after 1998 (Agostinho et al. 2007; Oliveira et al. 2020).

In addition, it is known that wetlands can sequester substantial amounts of carbon over time due to their high primary productivity and slow decomposition rates (Valach et al. 2021). Studies indicate that changes in water levels along with several localized components, such as the design of restoration projects, patterns in disturbance and succession, former land usage, and the effect of management strategies could have a significant effect on the yearly net carbon balance of wetlands (Luyssaert et al. 2007; Abbott et al. 2019). Thus, the protection of these ecosystems may provide a reduction in atmospheric CO_2_, thereby supporting climate change mitigation (Griscom et al. 2017).

In this context, the IVRP is one of the few Brazilian PAs designed to protect a freshwater ecosystem since most of them focus on protecting terrestrial biota and landscapes (Azevedo-Santos et al. 2018; Bailly et al. 2021). Thus, the use of freshwater environments only as the boundaries of terrestrial PAs, or protection through their casual incorporation into the terrestrial PA network, limits the ability to conserve aquatic biota (Azevedo-Santos et al. 2018, 2020; Bailly et al. 2021). Moreover, freshwater ecosystems are among the most vulnerable and human-altered ecosystems in the world (Malekmohammadi and Jahanishakib 2017), especially in developing countries. Furthermore, like many other PAs, the IVRP PA spatially matches with refugia areas for many species of fauna and flora in the face of future climate change (Ruaro et al. 2019; Bailly et al. 2021). Therefore, following the IVRP example, PA planning should explicitly incorporate freshwater ecosystems (Azevedo-Santos et al. 2018, 2020). Furthermore, existing PAs should receive sufficient funding (Silva et al. 2021) compatible with the crucial role they play in regulating climate, providing fishing and water resources, sustaining crop production, facilitating recreation activities, and enhancing biodiversity conservation (Malekmohammadi and Jahanishakib 2017; Bailly et al. 2021).

Brazilian PAs have recently been experiencing several environmental setbacks, both through direct actions on the environment and indirectly through changes in legal frameworks (Jesus et al. 2020; Moretti et al. 2020). An example of this is the ongoing inappropriate political decisions that threaten and question the effectiveness of PAs (Azevedo-Santos et al. 2017; Lima et al. 2020; Conceição et al. 2022), thereby undermining conservation efforts. In fact, these harmful decisions, which are emphatically opposed by the scientific community, frequently benefit unrestricted agricultural expansion and respond to lobbying or electoral support interests all of which occur at the cost of severe environmental impacts (Azevedo-Santos et al. 2017; Abessa et al. 2019; Alves et al. 2019). In this context, our work encompasses data from 1989 to 2018 and depicts the effects of several unsuitable political decisions over this period. The most recent attacks on the Brazilian environmental framework, especially during the presidential administration from 2018 to 2022 (Lima et al. 2020; Moretti et al. 2020), have yet to have their long-term impacts assessed.

Despite the recent misinformation and misguided policies, our results indicate that the land-sparing strategy adopted in the analyzed IPA appeared to be effective in protecting vulnerable ecosystems from anthropic expansion, while the SUA experienced an increase in farming activities along with the recovery of forest coverage through a land-sharing approach. Thus, despite the increasing level of anthropization in the SUA, its main goal to make nature conservation compatible with the sustainable use of part of its natural resources is being achieved (IUCN 1994). Additionally, to further enhance PA effectiveness, political actions should aim to resolve the problem of the severe underfunding of PAs (Silva et al. 2021). Also, enhancing the capacity for sampling and inventorying biodiversity in PAs may result in a considerable increase in biodiversity protection (Oliveira et al. 2017). Additionally, the effect of external impacts such as dams, which reduce water surface and wetland areas (Agostinho et al. 2007; Oliveira et al. 2020), should also be investigated.

Understanding PA dynamics can provide valuable knowledge for developing management plans and public policies that aim to protect biodiversity and improve social well-being (e.g., via the direct transfer of resources to conservation actions or payment for ecosystem services (PES)). In this context, the REDD+ mechanism, which encourages policies that consider the reduction of greenhouse gas emissions and an increase in forest carbon stocks (UNFCCC 2023), is an example of the potential of forest protection to generate financial income (Vongvisouk et al. 2016). Within the PAs of the Upper Paraná River, many municipalities receive money from the Brazilian Ecological Tax (ICMS Ecológico), which is a form of PES that aims to transfer financial resources to municipalities or their neighboring areas containing PAs (Brazilian Law No. 59/1991). However, those who receive it are not obliged to spend these resources on environmental causes.

The Brazilian Ecological Tax plan, which began in 1991 in Paraná state, is now present in 17 Brazilian states and at least one-third of all municipalities mationwide (SOS Mata Atlântica 2019). This major environmental initiative, which was first taken as a compensatory measure, now serves a role in encouraging the conservation of biodiversity (Loureiro 2002). In Paraná state, the number of municipal parks has tripled in the first 10 years of the plan (1991–2000), while the PA in municipal parks has increased by approximately 23,000 ha, representing a growth of nearly 1600% (Loureiro 2002; SOS Mata Atlântica 2019). In addition, in some more impoverished municipalities, creating a PA and receiving resources from the Brazilian Ecological Tax is more lucrative than performing other economic activities in the same region (Loureiro 2002).

We suggest that the outcomes of receiving the Brazilian Ecological Tax could be improved even further if part of the received financial resource is invested in both the PA’s funding and the municipality’s environment sector. Objectively, the Brazilian Ecological Tax is a successful PES measure that effectively promotes nature conservation and could be used as a model for the implementation of similar programs worldwide. In future approaches, it is important to consider that when dealing with PES programs and PA effectiveness, each scenario should be analyzed for its case-specific conditions, limitations, and potential.

## Conclusion

In summary, despite the increasing anthropization of the landscape, PAs seem to relieve the general process of change and protect natural categories, especially from agricultural expansion. The degree of protection of PAs also served an essential role in the main transition processes, with stricter protection leading to less human-induced changes in the landscape. Furthermore, we observed that the more restricted PAs have a lower rate of anthropic changes in the landscape, while the less restrictive PAs show the opposite trend. This latest trend may be the result of the inefficiency or insufficiency of land-use restrictions of this type of PA to contain anthropogenic changes in the environment, or the result of a leakage spillover effect, or both. However, new studies are needed to investigate these causes in greater detail. Despite this, both analyzed PAs seemed to achieve their objectives (i.e., being solely safeguarding nature or conciliating this protection with the human occupation of the landscape).

Finally, assessing PA effectiveness or its actual amount of protection remains challenging for researchers in many knowledge areas. Despite being a challenge, this knowledge is fundamental to avoid misunderstandings or poor policy decisions that could harm the environment. A closer dialog between the PA-adjacent communities, scientists, and decision makers is necessary to enhance the understanding of PAs so that science-based measures can be developed to improve environmental conservation, social welfare, and economic prosperity.

## Supplementary Information

Below is the link to the electronic supplementary material.

Supplementary file1 (PDF 529 KB)
