# Supplementary material for: Assessing the role of protected areas in the land-use change dynamics of a biodiversity hotspot
Source: Ambio. 2023 Jun 1;52(10):1603–17. doi: 10.1007/s13280-023-01886-5 (PMC10460765; doi:10.1007/s13280-023-01886-5)

Ambio

Supplementary Information

This supplementary information has not been peer reviewed.

Title: Assessing the role of protected areas in the land use change dynamics of a biodiversity hotspot

S1. Variation of the population within the study area between 1990 and 2010. Data obtained through the Brazilian Institute of Geography and Statistics (IBGE, 2023).

Data were obtained from the Brazilian demographic survey, carried out by the Brazilian Institute of Geography and Statistics between the years 1990 and 2010 (IBGE, 2023). Information was used at the census sector level, which corresponds to the neighborhoods, thus, all the municipalities that are contained within the boundaries of the PAs and buffer zone were analyzed. Based on the information for each municipality, a general sum was made to estimate the total population within the study area for each year.

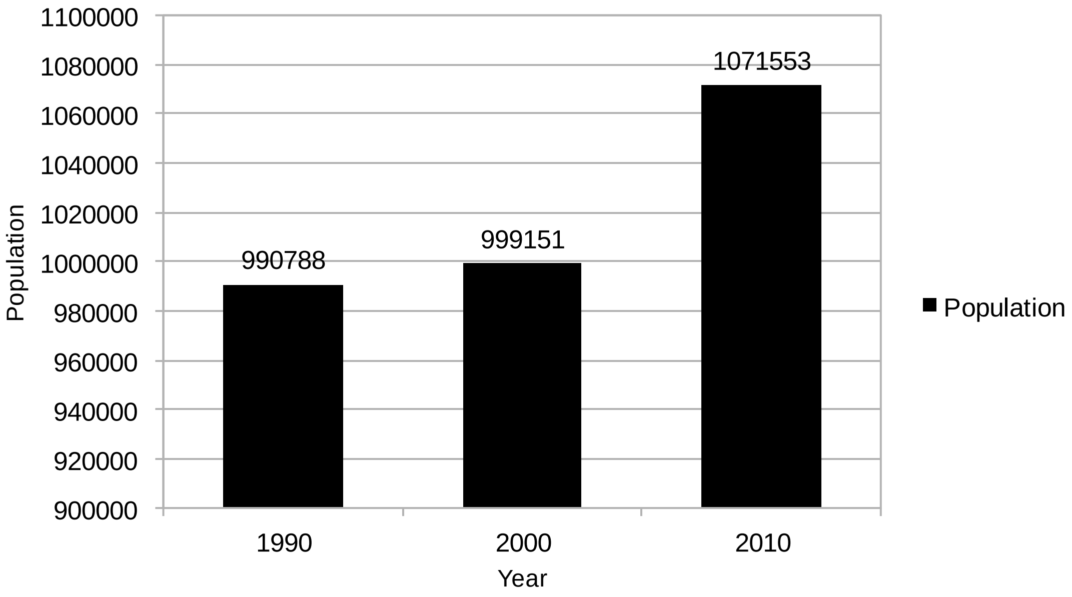

IBGE – Instituto Brasileiro de Geografia e Estatística. 2023. Downloads e estatísticas. Available at: https://www.ibge.gov.br/estatisticas/downloads-estatisticas.html Access 02/2023.

S2. Bar plots of the accumulated transitions during the periods of 1989–1998 (light gray), 1999–2008 (regular gray) and 2009–2018 (dark gray) for the Parque Estadual das Várzeas do Rio Ivinhema (PEVRI) and Parque Nacional de Ilha Grande (PNIG) areas.

PEVRI

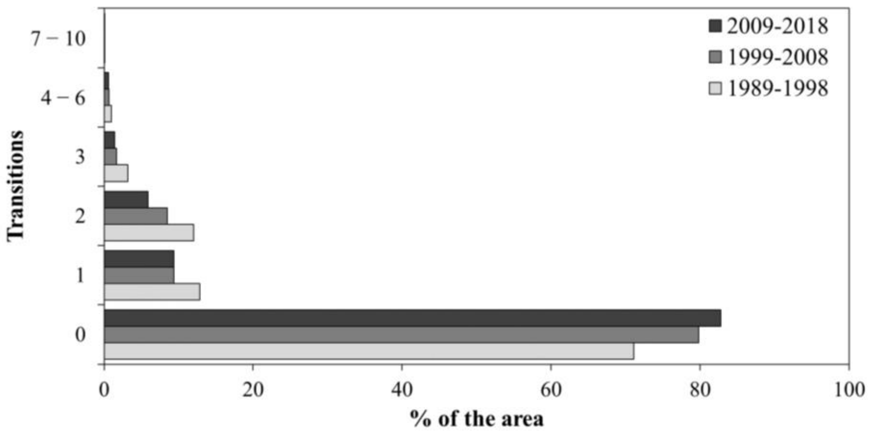

PNIG

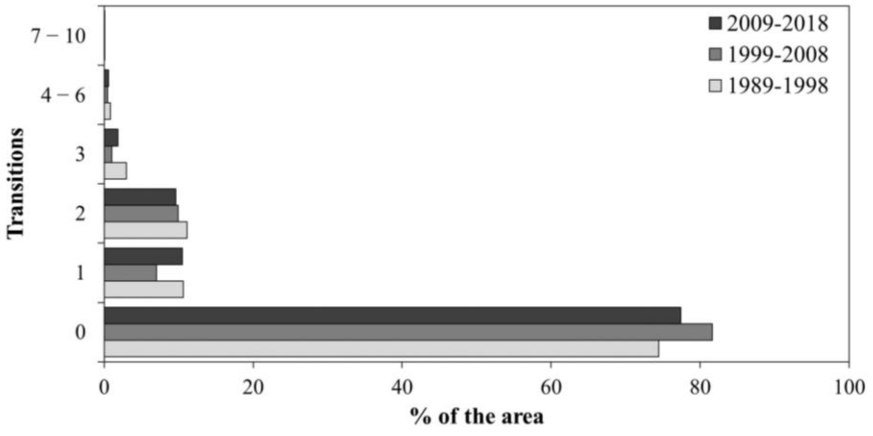

S3. Net gains (green), losses (red) and gross change (gray) of the land use categories for the Parque Estadual das Várzeas do Rio Ivinhema (PEVRI) and Parque Nacional de Ilha Grande (PNIG) areas, during the three intervals: 1989–1998 (left plot), 1999–2008 (center plot) and 2009–2018 (right plot).

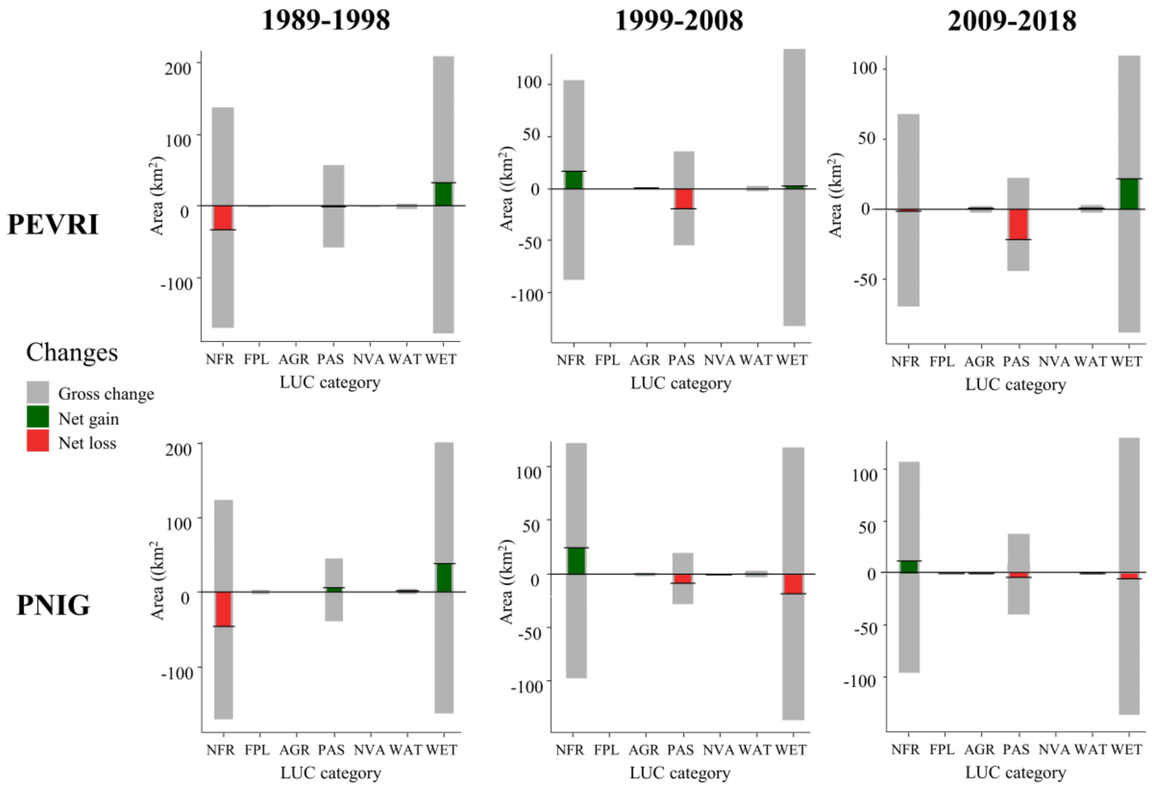

S4. Change intensity maps with the spatialization of the land use transitions between 1989 and 2018 in the study area.

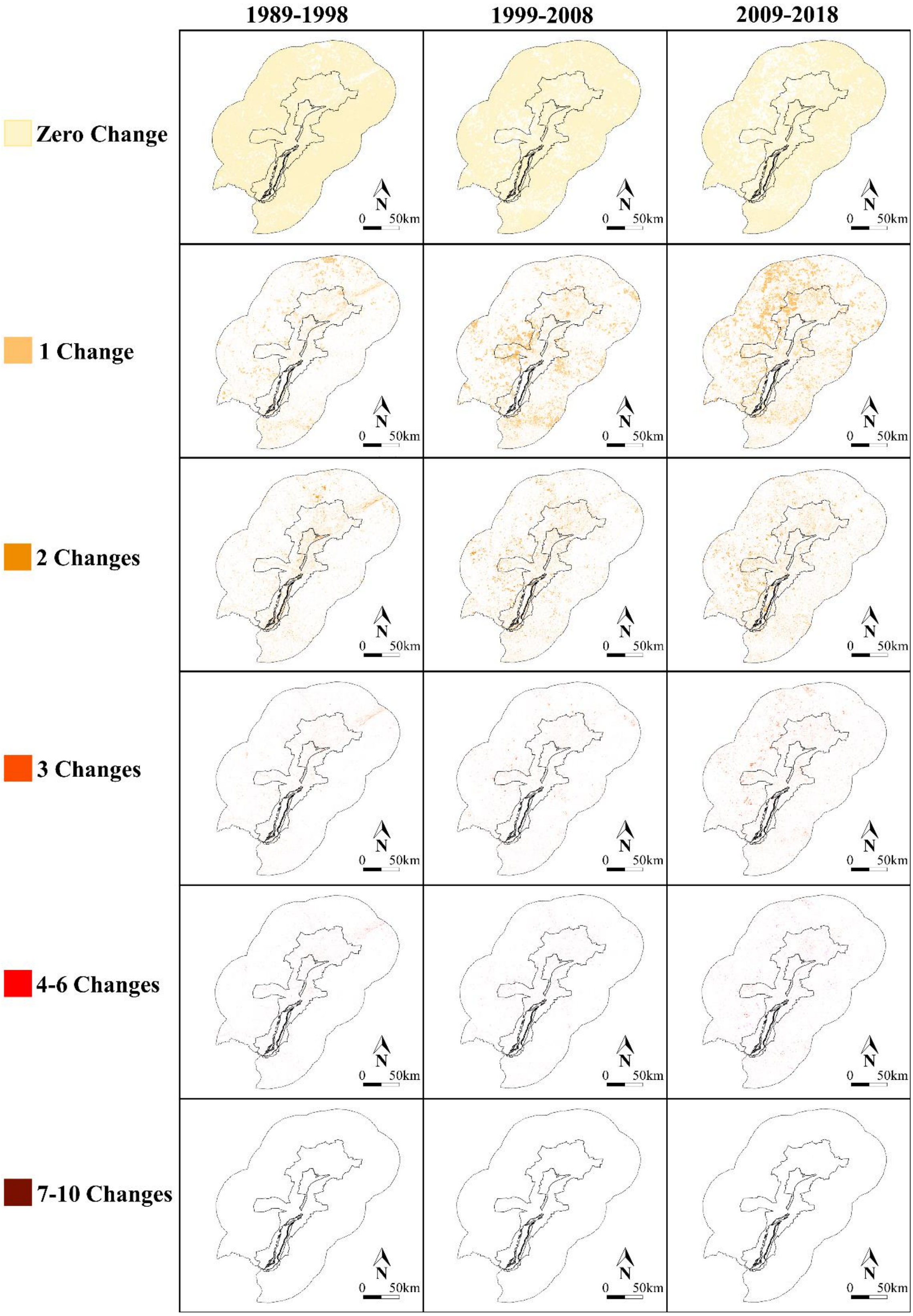

Supplement: Supplementary file 1 — Supplementary file1 (PDF 529 KB) [file 13280_2023_1886_MOESM1_ESM.pdf]
